# Improving influenza vaccine uptake in clinical risk groups: patient, provider and commissioner perspectives on the acceptability and feasibility of expanding delivery pathways in England

**DOI:** 10.1136/bmjph-2024-000929

**Published:** 2024-06-28

**Authors:** Ben Kasstan, Rajeka Lazarus, Ifra Ali, Sandra Mounier-Jack

**Affiliations:** 1 Department of Global Health and Development, London School of Hygiene & Tropical Medicine, London, UK; 2 The Vaccine Centre, Department of Global Health and Development, London School of Hygiene & Tropical Medicine, London, UK; 3 University Hospitals Bristol and Weston NHS Foundation Trust, Bristol, UK; 4 Severn Pathology, United Kingdom Health Security Agency, Southmead Hospital, Bristol, UK; 5 University of Bristol Medical School, Bristol, UK

**Keywords:** Public Health, Primary Prevention, Community Health

## Abstract

**Background:**

People under the age of 65 in clinical risk groups are at increased risk of severe complications and death from influenza. In England, influenza vaccine coverage rates in this cohort remain profoundly low. This qualitative study aimed to explore (1) the reasons that underly suboptimal influenza vaccine uptake among different clinical risk groups in England and (2) how healthcare providers and commissioners perceive the feasibility and acceptability of integrating the influenza vaccine programme in non-primary care settings.

**Methods:**

The study consisted of two phases. Phase I involved 32 semi-structured interviews conducted with individuals from three clinical risk groups: diabetes, chronic liver disease or chronic respiratory disease (or comorbidities). Phase II consisted of semi-structured interviews with 50 healthcare providers based in National Health Service primary and secondary care settings, and influenza vaccine commissioners and programme managers. Data were analysed thematically.

**Results:**

Access was not the primary issue underlying suboptimal vaccine uptake among participants in clinical risk groups, who instead cited low-risk perceptions of influenza infection and deficits of information about the relevance of vaccination for their condition management. Healthcare providers in non-primary care settings rarely discussed or recommended influenza vaccination across patient pathways, despite being able to address the concerns raised by participants in clinical risk groups. Healthcare providers were positive about the potential to offer vaccine recommendations and delivery, but questions remain around feasibility.

**Conclusion:**

Patient pathways are punctuated with varying opportunities to discuss or deliver influenza vaccines during the winter season, though the commissioning and organisation of chronic disease management shapes how clinical risk groups interface with primary/secondary tiers of healthcare services. Embedding vaccine delivery in non-primary care settings may help to reduce inequalities and offer patients at risk the information and consent pathways they desire but is not a cost-neutral innovation and requires resource allocation.

**WHAT IS ALREADY KNOWN ON THIS TOPIC**

- Rates of influenza vaccine coverage are low among people aged under 65 in clinical risk groups in England, though evidence suggests that the likelihood of accepting vaccination increases with the frequency of contact with healthcare providers. Closer attention to how and where people in at-risk groups interface with health services can then inform attempts to enhance influenza vaccine programme delivery to this cohort.

**WHAT THIS STUDY ADDS**

- Non-primary care pathways in England can be more effectively leveraged to recommend or opportunistically offer influenza vaccines to people in at-risk cohorts. Commissioning frameworks and performance-based incentives affect how clinical risk groups interface with healthcare services, which raises implications for how influenza vaccines are recommended or offered to at-risk cohorts in non-primary care settings.

**HOW THIS STUDY MIGHT AFFECT RESEARCH, PRACTICE OR POLICY**

- Health commissioners need to carefully assess the most acceptable and feasible opportunities to integrate influenza vaccine recommendations and delivery in non-primary care settings to effectively ‘make every contact count.’

## Introduction

 Influenza is a major public health issue that leads to approximately 500 000 deaths worldwide and up to 50 000 hospitalisations in England each year.^1^,^3^ The seasonal influenza vaccine programme aims to protect those most at risk from infection and to relieve pressure on healthcare services during winter seasons.^4^ People under 65 years of age living with long-term conditions (henceforth ‘clinical risk groups’) are at risk of severe complications, hospitalisation and death from influenza and are eligible to receive influenza vaccines free-of-charge under the National Health Service (NHS).^4^,^6^ England’s chief medical officer set a target of reaching or exceeding 60% influenza vaccine coverage in this cohort in 2011–2012,^7^ rising to 75% by 2013–2014 in line with the national and WHO target for the age 65+ cohort. Yet, coverage rates among clinical risk groups in England remain suboptimal and have been characterised by modest fluctuation since the onset of the COVID-19 pandemic (table 1).

**Table 1:** Influenza vaccine overage in years since the onset of the COVID-19 pandemic

Influenza vaccine uptake among people aged under 65 with one or more clinical risk factors (excluding healthy pregnant women and carers), based on uptake in GP patients in England.	
Year	% uptake
2022–2023	49.1
2021–2022	52.9
2020–2021	53
2019–2020	44.9

GPgeneral practice

Just 49.1% of people in clinical risk groups received an influenza vaccine in England in 2022–2023 and uptake across eligible clinical risk groups varied considerably (table 2).^8^ Coverage was highest among people living with diabetes (60.1%) and lowest in chronic liver disease (44.6% (henceforth CLD)).^8^ Mortality rates from influenza vary among clinical risk groups and are higher among people living with CLD (15.8 per 100 000) compared with diabetes (2.2 per 100 000).^5^

**Table 2:** Influenza vaccine overage rates in eligible risk groups, 2022–2023^8^

2022–2023 Influenza vaccine uptake among people aged 16 to under 65 with one or more clinical risk factors (excluding healthy pregnant women and carers), England.	
Clinical group	National-level coverage
Diabetes	60.3%
Chronic kidney disease	58.5%
Immunosuppresion	56.6%
Chronic neurological disease	54.9%
Chronic respiratory disease	52.4%
Asplenia	51.0%
Chronic heart disease	49.8%
Chronic liver disease	44.6%

The reasons that underlie suboptimal influenza vaccine uptake among clinical risk groups are complex. Past studies conducted in higher-income countries highlight the role of underlying social determinants of health, including deprivation, ethnicity, region of residence and inequalities in access to healthcare.^9^,^11^ Deficits in information and concerns of adverse reactions have also been reported.^12^ While there is no consensus on the most effective approaches to improving influenza vaccine uptake among clinical risk groups, there is evidence to suggest that the likelihood of vaccination increases with the frequency of contact with healthcare providers.^13^

The influenza vaccine programme in England is predominantly delivered via primary care.^4^ Initiatives to improve access have included flexible general practice (GP) surgery hours and delivery through community pharmacies.^14^ GP-level coverage rates have been improved by strengthening leadership over influenza vaccine delivery, for example, by conducting service reviews to inform and motivate staff.^15^ While such delivery strategies may have helped to increase influenza vaccine uptake over the past 10 years,^16^ coverage rates in the under 65 at-risk cohort remain concerningly low (tables1^2^).^7 8^ In 2020, opportunistic vaccination in all secondary care settings was recommended to enhance access to vaccination.^17^ Co-ordination of COVID-19 and influenza vaccines has raised new opportunities to enhance programme delivery strategies in England and internationally, particularly through public communications as well as appraisals of how to effectively offer vaccines opportunistically.^18 19^ Yet, concerns have also been raised that ‘vaccine fatigue’ and public doubt in COVID-19 vaccine effectiveness may be directed to routine programmes.^19^

Against this backdrop, the aims of this study were to explore (1) the reasons underlying suboptimal influenza vaccine uptake among different clinical risk groups and (2) how NHS healthcare providers (HCPs) and commissioners perceive the feasibility and acceptability of integrating the influenza vaccine programme in non-primary care settings.

## Methods

A two-phase qualitative study design was devised. Phase I involved 32 semi-structured interviews conducted with individuals in 3 clinical risk groups. Phase II consisted of semi-structured interviews with 51 HCPs based in NHS primary and secondary care settings, and influenza vaccine commissioners and programme managers. We sought to interview a minimum of 30 participants from the phase I cohort and 25 from the phase II cohort to enable in-depth analysis of data according to clinical risk groups and healthcare provision (and commissioning). Further particulars about sampling and recruitment are outlined below.

### Phase I: clinical risk groups

Clinical risk groups were selected based on disparities between the highest (diabetes) and lowest (CLD) levels of vaccine coverage. Due to recruitment challenges, inclusion was extended to chronic respiratory disease (CRD)—the clinical risk group with the largest number of registered patients under 65 years of age.^8^ Participants were eligible for inclusion if they were: aged between 18 and 64 years; resident in England and Wales; had missed at least one influenza vaccine in the previous 3 years (2019–2020/2020–2021/2021–2022) and had a confirmed diagnosis of diabetes, CLD (eg, chronic viral hepatitis) or CRD (eg, chronic obstructive pulmonary disorder (COPD), asthma). Participants with comorbidities involving any of these conditions were eligible to participate in the study.

A significant number of participants had never received an influenza vaccine (n=8/25%), of whom most were from Black and Asian minority ethnic backgrounds (n=6/75%). Participant numbers per clinical risk group and participant characteristics are outlined in tables3^4^.

**Table 3:** Participant numbers per clinical risk group

Clinical risk group	Number of participants
Diabetes	16
COPD/asthma	9
Chronic liver disease	3
Comorbidity	4
Total	32

COPDchronic obstructive pulmonary disorder

**Table 4:** Phase 1 participant characteristics

Phase 1 participant no.	Clinical risk group	Residence (by NHS Region)	Age	Sex	Ethnicity	Recruitment route
1	T1D	South West	55	F	White British	Referral
2	T1D	South West	58	F	White Scottish	Facebook support group
3	T1D	South West	42	M	Black African	Referral
4	T1D	London	32	M	Other	Referral
5	T1D	London	30	M	Black African	Referral
6	T2D	London	28	M	Black Caribbean	Referral
7	T2D	London	28	F	Other	Social media
8	T1D	London	29	M	Black Caribbean	Social media
9	T2D	London	32	M	Other	Referral
10	T2D	South West	38	M	Mixed (not specified)	Referral
11	T1D	London	30	M	Other	Referral
12	T1D	London	50	F	White British	Diabetes Support group
13	CLD	Wales	62	F	White Welsh	Facebook support group
14	T1D	South East	47	F	White British	Facebook post
15	T2D	North West	34	F	White British	Recruitment agency
16	T2D	East of England	47	F	Asian- Indian	Recruitment agency
17	Co-morbidities: T2D; CLD	North East & Yorkshire	43	M	White British	Recruitment agency
18	Asthma	London	27	M	White British	Recruitment agency
19	T1D	East of England	47	F	White British	Recruitment agency
20	Asthma	London	39	M	Mixed (English/Jamaican)	Recruitment agency
21	COPD	London	40	F	British Asian	Recruitment agency
22	CLD	East of England	53	F	White British	Recruitment agency
23	CLD	North East & Yorkshire	60	F	White British	Recruitment agency
24	Comorbidities: CLD; Asthma	London	59	F	White British	Recruitment agency
25	Asthma	London	39	F	Black British	**Recruitment agency**
26	COPD	East of England	59	F	White British	Recruitment agency
27	Comorbidities: Asthma; T2D	London	32	F	British Bangladeshi	Diabetes self-management meeting
28	Asthma and COPD	London	41	F	African Caribbean	Recruitment agency
29	Asthma	London	30	M	African Caribbean	Recruitment agency
30	Asthma	East of England	47	F	White British	Recruitment agency
31	Comorbidities: asthma; T2D	East of England	37	F	White British	Recruitment agency
32	Asthma	London	30	M	British Asian Indian	Recruitment agency

CLDchronic liver diseaseCOPDchronic obstructive pulmonary disorderNHSNational Health ServiceT2Dtype 2 diabetes

Participants were recruited via advertising on social media platforms, health charities and a market research recruitment agency. Phase I participants received a £20 voucher as reimbursement for their time.

### Phase II: HCPs and commissioners

Primary care HCP included GP surgery teams, community care (eg, diabetic eye screening and self-management services) and community pharmacy. Secondary care HCPs were based in hospital services for CRD and CLD, diabetes, infectious diseases, or pharmacy teams. Vaccine programme managers or commissioners were based in NHS England and London Region, Integrated Care Boards or local authority public health teams (see table 5). Community pharmacy data were drawn from NHS commissioning teams. All phase II participants provided services in either London or Bristol. Participants are coded according to primary care; secondary care; influenza vaccine managers and commissioners; linked professionals (eg, commissioning teams beyond vaccination).

**Table 5:** Numbers of phase II participants recruited

Role	London	Bristol	Total
Clinicians (diabetes)	0	1	1
Clinicians (chronic liver disease)	3	2	5
Clinicians (respiratory/infectious diseases/renal)	2	1	3
Diabetes specialist nurses	1	1	2
Liver disease specialist nurses	0	2	2
Specialist nurses (other)	0	1	1
Hospital pharmacists	3	1	4
General practitioners	4	2	6
Practice surgery nurses (including advanced or lead nurse practitioners, district nurses)	6	0	6
Practice managers	3	0	3
Community diabetes teams (retinal screening, self-management, specialist diabetes community nurse)	4	0	4
Influenza vaccination leads (commissioning/management)	5	2	7
Local authority public health teams	5	1	6
Pharmacy advisor/community pharmacist	2	0	2
Total	38	14	51

Participants were recruited via professional networks, by direct invitation, past research studies, snowball sampling methods, and clinical networks and associations (British Association for the Study of the Liver; the National Institute for Clinical Excellence Clinical Research Network).

### Data collection and analysis

All interviews were conducted in-person or via telephone or software between February and November 2023. Phase I and II participants provided informed consent (written or oral) to take part in the study. Interviews lasted between 30 and 40 min and were recorded and notes made with permission (online supplemental material 1). Interviews were transcribed using online transcription service (Otter.ai) and anonymised using codes (table 6).

**Table 6:** Participant code key

Participant code key		
Phase number 1 (P1)	Participant number	Condition Asthma Chronic obstructive pulmonary disorder Type 1 diabetes (T1D) T2D Chronic liver disease
Phase number 2 (P2)	Primary care participant number Secondary care participant number Linked professional participant number Vaccine commissioning/manager participant number	Role for example, GP Nurse Consultant Hospital pharmacist Pharmacy advisor

GPgeneral practice

Consistent with approaches in qualitative research,^20^ sampling was determined by the research team based on achieving data saturation (emergence of no new concepts). Separate topic guides were designed for phase I participants and phase II participant groups and were informed by existing literature (online supplemental material 2). Guides explored experiences surrounding influenza vaccines, perceived acceptability of integrating vaccine delivery in non-primary care settings and lessons learnt from COVID-19 vaccine programme delivery. Key analytical themes were drawn directly from the data through a grounded theory approach.^20^ Emergent coding themes were reviewed and discussed extensively between the research team and refined as the results of these discussions. Data were coded and managed using software (NVivo and Excel) following steps outlined in applied social science studies.^21^

### Public and patient involvement

On 3 October 2022, the NIHR HPRU Vaccines and Immunisation hosted a public and patient involvement event to ‘Help Shape Research in Vaccines and Immunisation.’ The event was designed to elicit views of both patients in clinical groups, and HCP involved in their care, on their attitudes and access towards influenza vaccination. Responses from HCP and people in clinical risk groups were noted to inform the topic guides, based on their experience of delivering or receiving influenza vaccines. Attendees were not involved in the study recruitment, though we approached Diabetes UK to promote the study in patient group networks. Results have been shared in summary form with Diabetes UK, the British Association for the Study of the Liver and NHS England.

## Results

Results are reported in two parts: reasons for suboptimal influenza vaccine uptake among clinical risk groups; and HCP and commissioner perspectives on integrating vaccine programme delivery in non-primary care settings. These two overarching themes are closely interlinked as key concerns raised by participants in clinical risk groups centred on the lack of condition-specific engagement about risk from influenza, which was reflected in the general absence of conversations around vaccination across patient pathways.

### Reasons for suboptimal vaccine uptake

Key reasons underlying suboptimal vaccine uptake include influenza risk perceptions, tolerating and trusting influenza vaccines, and information availability.

#### Influenza risk perceptions

Participants across clinical risk groups acknowledged that influenza causes severe complications for people living with long-term conditions and age-related risk:

So, I think those who have more conditions relating to immunity and breathing may be better candidates. But I’ve just got a perception in my mind that it’ s for really old people or people that are like, very, very at risk*.* (P1_18_Asthma).

Yet, participants often did not feel they were personally ‘at risk’—particularly if they felt confident to manage their long-term conditions effectively:

I understand that I've got a respiratory condition. But at the same time, if I'm managing it, I know how to work with it*.* (P1_25_Asthma)

Conversely, HCP in secondary care felt that people with long-term conditions were not aware how their condition placed them at increased risk of serious illness from influenza:

What they don’t realise is by having abnormal glucose metabolism, they’re actually more vulnerable to getting viral and bacterial infections. (P2_SC6_Consultant_Diabetes)

Influenza vaccination was generally viewed as irrelevant against the backdrop of low-risk perceptions:

I think there was something about just not feeling it was necessary*.* (P1_14_T1D)

While HCP viewed patient risk perceptions as a key barrier to vaccine uptake, they also recognised that people aged under 65 are of working age and required convenience in access:

They might clinically be at risk but perhaps don’ t realize that flu can be damaging to their health […] the working population are mobile, but they’re also somewhat time-bound, because they can’t come as flexibly as perhaps people who are retired. I think the more access sites, the easier it will be for them to get to it*.* (P2_PC13_GP)

#### Tolerating and trusting influenza vaccines

The risk of side effects (or immune responses) while managing long-term conditions was a key concern for participants across clinical risk groups:

The side effects are a massive issue for me. It’s like we can fix this one thing, and there’s 10 other things that may happen as we fix this. (P1_20_Asthma)

This was particularly the case if participants felt vaccination could cause problems for chronic disease management:

I was frightened of having a flare up of my symptoms. That was the main concern really. (P1_13_CLD)

Experience of side effects or immune responses following influenza vaccination in previous years deterred subsequent uptake:

I was really poorly after the flu jab. And then I thought, “oh, whatever” and brushed it off. The following year comes round and I took it again. I was really poorly. It took me so long to recover from it. I ended up having to have steroids post-flu jab and I just made a commitment that I'm never having the flu jab again, ever. It just made me so, so ill. (P1_30_Asthma)

HCP reported misconceptions around side effects and vaccine effectiveness among patients, highlighting information needs among clinical risk groups:

There’s the classic, “I've had it one time, and it gave me the flu.” So, we really try to explain that this vaccination is inactivated. It can't do that. It was perhaps coincidence that you did develop the flu after it last time. (P2_PC3_Nurse)

#### Information availability

Participants were aware that they were eligible and encouraged to receive influenza vaccines, predominantly because of notifications (usually generic text messages or letters) from their GP surgeries or when learning about their conditions:

If you’ve got liver disease, you’re very much encouraged to take the flu vaccine up. (P1_13_CLD)

Invitations from GP surgeries did not convey a needs assessment for vaccination (ie, being at risk of severe illness):

Yeah, I get text reminders from them every year […] it’s almost a bit of a numbers game to them, that they have to obviously get as many people vaccinated as possible. That’s their target. (P1_12_T1D)

Similarly, participants in clinical risk groups consistently reported a lack of tailored information detailing the importance of influenza vaccination for people with long-term conditions, which prevented them from feeling able to make informed health decisions:

It’s absolutely terrible. There’s no information, no communication, whatsoever on the flu vaccine and I feel really do feel like we're left out, especially people with health conditions, the younger generation. It’s more targeted to the old generation getting it done, rather than the younger people, we're obviously just like pushed aside. You'll get a text message, “Oh, you got to come in.” That’s it. (P1_31_Asthma/T2D)

Participants across clinical risk groups valued information that accurately conveyed the risk of severe complications from influenza and the benefits of vaccination for people with long-term conditions, without resorting to scare tactics:

It needs to be presented in a non-fear, non-scary way as much as possible. Just to make people aware from a medical basis to make an informed decision, rather than, a Doctor saying, “you should take your flu vaccine.” (P1_12_T1D)

HCP agreed that education and information around vaccination was critical to improving uptake among clinical risk groups, but that GP surgery teams were not able to provide this guidance on a one-to-one basis because of competing demands:

It’s difficult to ask clinicians to do any more. We just don't really have the time. We would probably see two patients a day if we did everything to best practice. If we were to explain everything about diabetes, and everything about a particular 10 drugs that our patients are on or whatever*.* (P2_PC6_Nurse)

### Perspectives on expanding vaccine delivery pathways for clinical risk groups

GP surgery teams identified limitations around engaging clinical risk groups with influenza vaccination and opportunistic delivery due to capacity. Patient groups and HCP reported that vaccination was rarely discussed or delivered in secondary care settings, and while delivery in secondary care was acceptable in theory, practical questions around feasibility remained.

#### Limitations on vaccine delivery in GP surgeries

Participants were aware that influenza vaccines can be accessed via GP surgeries and community pharmacies, and cited examples of convenient GP clinic arrangements (eg, evenings and weekends). The general view was that access was not a key issue:

It’s pretty easy to do, pretty easy to book, pretty easy to get and have. (P1_17_T2D)

Possibilities to have informed discussions about influenza vaccines or to be vaccinated opportunistically were limited:

When you go to the doctors, they go on the computer and say, “you do realize your due for your flu vaccine” And they don’t say, “well, I'll give it to you now.” They say, “make an appointment with the nurse.” This is what annoys me*.* (P1_26_COPD)

GP surgery teams described proactively offering vaccines opportunistically to patients with long-term conditions, though one practice nurse noted how limitations on orders during the winter season presented consequences:

This season just gone has been quite frustrating because we’ve just had the Saturday clinics. We’ve been told “right, don’t use any vaccines opportunistically to start off with because we need to know we've got all the vaccines for them booked in” […] But I think we missed out because you can get them opportunistically*.* (P2_PC15_Nurse)

More broadly, GPs described how limited capacity meant that they felt unable to consistently offer influenza vaccines opportunistically to eligible patients:

If you’re busy, and you’re running late, the last thing on your mind is to give a flu vaccine. They [staff] have to get through a whole host of things in that [annual] review. So, to add on to that? Would they have enough time to administer the vaccine? I'm not sure. (P2_PC5_GP)

Strained GP capacity during winter seasons then had an impact on the ability to ‘make every contact count’ and offer influenza vaccines opportunistically. Amidst these constraints, GP surgery teams reported being more likely to recommend influenza vaccination or offer vaccines opportunistically to patients in particular clinical risk groups:

I think the people with a respiratory diagnosis, you're much more likely to then stress the point about that for them, rather than diabetes […] And perhaps I think, for me personally, but I think otherwise in the practice, I've seen that as well, that fewer people would consider that immediately for diabetic patients. (P2_PC1_GP)

#### Acceptability of expanding delivery pathways

HCP signalled that there is significant potential to improve how the NHS engages clinical risk groups with the influenza vaccine programme, and how delivery is embedded in patient pathways.

Diabetes care teams explained various touchpoints where it would be acceptable and appropriate to engage patients with influenza vaccination, even if this was not current practice. The diabetes patient pathway includes primary care (regular GP appointments and an annual review), but also management through community care (annual diabetic eye screening appointment; possible engagement with a dietitian, podiatrist, dialysis centre; self-management and education programme). HCP cited how self-management and education services were important for patients with recent diagnoses, yet rarely recommended vaccination:

Well, they [vaccine conversations] don't [happen]. […] we have some patients who have only recently been diagnosed with diabetes, so they're only just getting grips to having diabetes and what that means for them, and what changes they need to make. They need to have had diabetes for three months to attend our courses. So, I'd say that population of patients, yes, might be more and less knowledgeable about how not having a vaccination impacts them and how the flu if they get it, for example, makes them more vulnerable […] I don't think there’s a barrier [to discussing vaccination], it’s just something that we haven't really done*.* (P2_PC7_DiabetesTeam)

Consequently, opportunities were missed to engage patients with the importance of influenza vaccination at a formative point in their care pathway. HCP consistently highlighted that discussing or delivering vaccines across patient pathways was not part of current practice but indicated the benefits of integrating vaccine delivery in spaces where patient demand for services was high:

We have a fantastic diabetic retinal screening programme […] most of our patients do attend for their retinal screening, but there is no mechanism for a patient to get their flu vaccination if they were attending that appointment. Because diabetic retinal screeners don't do flu vaccinations. (P2_PC6_Nurse)

People living with conditions linked to CLD are more likely to receive care from specialist hospital services, including 6-monthly ultrasound surveillance and tumour marker blood tests, clinical assessments every 6–12 months and outpatient clinic appointments. Hepatologists considered integrating influenza vaccine delivery in existing outpatient pathways to be feasible and strategic during winter seasons and suggested that this approach may help to reduce inequalities in vaccination among people from ethnic minority backgrounds:

Every 6 months, a patient with CLD should have an ultrasound scan as part of cirrhosis monitoring and liver cancer surveillance. So, by definition, one of those is going to be around the sort of time where it would not be unreasonable to have a flu vaccine. So that’s everybody with cirrhosis. That’s everybody with hepatitis B who has a family history of liver cancer. People who are, Asian men over the age of 40, Asian women over the age of 50, and all Africans over the age of 20. So that’s probably the best point of intervention. (P2_SC1_Consultant_Hepatology)

While secondary care HCP rarely discussed or recommended influenza vaccination with patients during outpatients appointments, they were conscious that COVID-19 vaccines were routinely recommended during roll-out in 2020–21:

It [influenza vaccination] doesn't come up. That’s the reality. Sometimes I get asked, should I have the flu jab? And I'll say, “Yeah, of course you should.” Certainly, when it came to COVID, we were absolutely recommending COVID vaccination. (P2_SC1_Consultant_Hepatology)

HCP had clearly set precedents by routinely recommending vaccines to patients with long-term conditions under their care. Participants in clinical risk groups were open to integrated delivery pathways, but what mattered most was convenience and being vaccinated by trained HCP:

If they’re all trained healthcare professionals, no I’d have no issues with them doing it in any of those environments*.* (P1_17_CLD/T2D)

#### Feasibility of embedding vaccination in patient pathways

HCP viewed expanding influenza vaccine programme delivery in community care pathways such as retinal eye screening favourably, but questions around feasibility were raised due to limitations on space and capacity to administer vaccines to patients:

There is time for vaccination to be done, whilst they [patients] are waiting. I think it’s a very good and opportunistic approach to do it. The main issue here would be the space—where is it going to be done? Because usually there is minimal waiting area, there is minimal room available. Everybody is struggling for a little room. (P2_PC9_DiabeticEyeScreening)

The perceived feasibility of embedding vaccine delivery in non-primary care settings depended on an assessment of the most appropriate delivery points and who would have responsibility for vaccination. HCPs acknowledged that there were systems-level limitations which prevented them from vaccinating patients admitted to hospital but did not necessarily feel that delivery via wards was the most feasible or appropriate point to offer vaccination:

It’s been a constant frustration that you can see a patient who’s in your hospital, who’s not vaccinated, and to not actually have the means to vaccinate them. But it would be much better done at discharge. They're more aware of the conversations. Having a conversation where you're slightly out of it on the wards isn't going to necessarily stay with you. (P2_SC14_Consultant_InfectiousDiseases)

Acceptability and feasibility were, therefore, not always aligned. However, a respiratory consultant described in patients as a feasible site for delivery due to the authority of ward staff to prescribe and administer a range of pharmaceuticals:

The doctors prescribe and the ward nurses administer—they have been trained to, and are very used to giving other treatments, including injections, so you don’t need a specialist nurse or roving ‘vaccinator’ to come and do it—it just happens as part of “treatment” rounds. (P2_SC15_Consultant_Respiratory)

Offering influenza vaccination at the point of discharge in hospitals was considered by HCP to be most the most appropriate time, as patients were perceived to have improved since admission and were waiting to return home:

When we admit a patient, it might be for something quite trivial. If they’re really ill, it’s not the right time to vaccinate somebody. But many of them could be vaccinated opportunistically at the point of discharge. (P2_SC8_Consultant_Hepatology)

Outpatient clinics were perceived to be effective settings to reach a larger number of eligible patients with underlying long-term conditions due to back-to-back appointments:

If you had someone doing flu vaccines in our outpatient clinic, we have clinics running all the time, every day of the week, morning and afternoon, sitting doing flu vaccines. I'm sure you would catch quite a lot of people. Again, with the inpatients, we can certainly offer it, but again, if patients are in or such a short time, we would normally just send them to their GPs. I think outpatients would be a good place to catch patients if you wanted to administer it to them. (P2_SC7_Pharmacist)

HCP also perceived it feasible to have their patients signposted to vaccine clinics stationed by outpatient services and vaccinated before or during appointments:

If we had a vaccine clinic running alongside our clinic, so I could just say to the patient, “look, I really think you should go and get vaccinated. Pop in now before you go home,” or while they were waiting to see me somebody could have approached them and offered it would have made a world of difference. (P2_SC10_Consultant_Hepatology)

While delivery via hospitals was considered by programme managers to be costlier compared with primary care, participants recognised that integrated delivery approaches offered an opportunity for reducing expenditures by preventing hospital admissions:

It would be quite expensive compared to the number of vaccines you give. And that’s the problem here, isn't it? We're rewarded by volume. But actually, we said this the whole way through COVID, it’s crackers, because the one patient that you might get who’s got lots of co-morbidities, who was most likely to trip into a hospital bed if they get flu or COVID, that patient has value, should be valued at hundreds of pounds. (P2_VCM4)

Perceptions of feasibility then need to be understood in relation to the design of appropriate targets and incentives which focus on reducing inequalities in the immunisation system.

#### Enhancing vaccine recommendations

Outpatient clinic letters and summaries were rarely used by specialist clinicians as an opportunity to recommend influenza vaccination. Just one HCP included a checklist about vaccination status in clinic letters, which primary care teams could review with the patient:

So, my I have a clinical summary at the top of every letter, one of the items will be “had annual flu vaccination every year” or “doesn’t have,” or “has been recommended to have,” “has had Pneumovax up to date with COVID.” Every single clinic letter, and in the text, which is a letter written to the patient, there will be a discussion about vaccination either saying “you're fully vaccinated’ or ‘respect your wishes not to, however, as you know, would have been strongly recommended. (P2_SC15_Consultant_Respiratory)

GPs were positive about including vaccine recommendations in specialist clinic letters, which might reinforce recommendations in primary care and offer condition-specific information about risk:

It highlights the importance even more if the consultant is saying it too […] as extra evidence that it’s important they have it. (P2_PC13_GP)

Such letters might also offer a prompt for community pharmacy teams to discuss vaccination with people in risk groups:

As Commissioners, we can also send out communications to say, “if you receive a letter from a consultant the patient presents, please consider them for this option.” (P2_LP7_PharmacyAdvisor)

However, a secondary care HCP was concerned about the additional administrative burden and described the need for a pro forma that was simple to implement:

If there had been an automated rubric that I could just click and it would have appeared or had been automatically on the letter, yes, that would have made a big difference […] I would. But I would have seen it as being worthwhile, if it had been facilitated, because the text was already there. And it was just another click as opposed to going to have to type “I've mentioned to this patient, and they need that they would benefit from flu vaccination.” So, to say, “on the basis of their liver disease, they would benefit from flu vaccination and I recommend that they come and talk to you full stop.” (P2_SC10_Consultant_Hepatology)

## Discussion

Broadening delivery pathways has been a feature of programmatic change in recent years to benefit the under 65 at-risk cohort, who are of working age.^14 16^ Yet, access was not the primary barrier to vaccination for participants of this study. Participants were more concerned with feeling informed about the risks associated with influenza and relevance of vaccination for managing long-term conditions, which is consistent with past studies conducted in Europe and the USA.^1222^,^24^ Participants in patient groups tended to minimise the risks of influenza, yet held heightened concerns about vaccine safety and effectiveness. Consistent with social science research on public interactions with vaccinations,^25^,^28^ decisions were influenced by individual notions of health and evidence that diverge from public health positions. Opportunities to adjust risk perceptions, by receiving condition-specific information from their GP surgery or specialist teams were scant.

HCPs in non-primary care settings are well placed to offer relevant information but rarely discussed influenza vaccination with patients—despite previously recommending COVID-19 vaccination. HCPs in non-primary care settings often acknowledged that engaging clinical risk groups with the influenza vaccine programme was acceptable, and several clinical touchpoints were flagged as possible sites to introduce or offer vaccination to patients. However, concerns of feasibility often gravitated around logistics, resources and staff training. Concerns about patient condition influenced perspectives of delivery in hospital settings, with the point of discharge viewed as the most acceptable site to offer vaccination.

Previous studies highlight that the likelihood of influenza vaccination increases with the frequency of contact with healthcare professionals.^13^ GPs in this study would more proactively recommended vaccination to patients from certain risk groups than others. The commissioning of chronic disease services^29^ may also play a role in influencing the frequency of patient–provider contact, and conditions which are predominantly managed in primary care may benefit from more opportunities to receive or be recommended vaccinations. GP surgeries are expected to deliver a 100% vaccine offer to the eligible practice cohort as part of commissioning contracts.^30^ GP surgeries receive metric-based financial incentives to provide services for registered patients with type II diabetes, COPD and asthma under the Quality and Outcomes Framework (QOF).^31 32^ GP surgery teams might see patients with these long-term conditions more regularly for procedures that can be performed at primary care level, including an annual review to help manage long-term conditions (where vaccines may be discussed or offered). CLD is instead managed by hospital teams, perhaps because relevant procedures and results require specialist oversight. GPs are not incentivised under the QOF to conduct annual reviews with patients with CLD^33^ and hence do not have the same opportunities to maximise vaccine uptake among all eligible risk groups. Further evaluation is needed, however, to ascertain the links between service commissioning and disparities in vaccination coverage. As people living with CLD are more likely to engage with hospital teams as part of condition management, they might benefit more from offering vaccine recommendations and condition-specific information in specialist clinic letters.

Models of best practice to maximise vaccine uptake in clinical risk groups are available and may help to address HCP concerns around feasibility, but these resources appear to be siloed in medical specialties.^34^ ‘Getting it Right First Time’^34^ is a national programme to increase coverage and improve NHS care by reducing variation and inequalities in the health service. A case study for best practice cites how the Whittington Health NHS Trust introduced influenza vaccine delivery on respiratory wards as a ‘preventing future illness’ intervention.^34^ This model efficiently identified patients who had not received a vaccine, offered prescribing via dropdown menus and improved information sharing with GPs to ensure records were updated. Expanding such delivery models requires a clear understanding of the contextual issues that influence implementation within secondary care.

Vaccine recommendations from HCP are pivotal influences on decisions among people in clinical risk groups. Expanding and embedding influenza vaccine recommendations across patient pathways requires a commitment to training HCP in vaccine confidence and to underwrite the cost of regular training programmes. While HCPs in this study were positive about the potential to discuss or deliver influenza vaccines in non-primary care settings, it should be noted that influenza vaccine uptake levels among NHS staff in England have declined since the onset of the COVID-19 pandemic in 2020. Just 49.4% of all front-line HCPs with direct patient care received the seasonal influenza vaccine in 2022–2023 in England, significantly lower than uptake rates in 2021–2022 (60.5%).^35^ Reasons for influenza vaccine decline among HCP include low-risk perceptions.^36^,^38^ Future studies should assess links between HCP ambivalence towards the influenza vaccine and implications for programme delivery to eligible cohorts.

### Strengths and limitations

A strength of this study was the combination and selection of clinical risk groups and HCP across primary and secondary care tiers. Our approach indicates that the commissioning of chronic disease care in England may impact the frequency with which people engage with HCP and the opportunities to maximise how the influenza vaccine programme is integrated into patient pathways for clinical risk groups. Phase I participants were included in the study if they had missed one vaccine in the past 3 years, and 25% of participants had never been vaccinated against influenza. Hence, the large proportion of influenza vaccine decliners may have impacted the results—particularly concerning efficacy and trust in the programme. The research occurred in the context of COVID-19 pandemic recovery and may have raised positive and negative biases, as the challenges posed by the pandemic have raised lessons for health systems,^39^ but also issues of HCP burn-out^40^ that may affect acceptability of additional task-management and issues of vaccine fatigue in the general public.^19^

## Conclusion

Integrating influenza vaccine discussions and delivery pathways in hospital settings offers potential for improving uptake in the under 65 at-risk cohort, particularly among risk groups that are less likely to be managed in primary care. Patient touchpoints need to be assessed to identify the most effective site to engage patients in a way that is conducive to programmatic requirements around capacity, logistics and safety. HCPs recognised the benefits of engaging their patients with the influenza vaccine programme, but limitations around feasibility were cited. Evaluating current influenza vaccine delivery in secondary care settings, particularly at the point of discharge, may help to identify models of best practice that can be implemented more broadly.

## supplementary material

10.1136/bmjph-2024-000929 online supplemental file 1

10.1136/bmjph-2024-000929 online supplemental file 2

## Data Availability

No data are available.
