# Correction: Improving influenza vaccine uptake in clinical risk groups: patient, provider and commissioner perspectives on the acceptability and feasibility of expanding delivery pathways in England

**DOI:** 10.1136/bmjph-2024-000929corr1

**Published:** 2024-07-18

 Kasstan B, Lazarus R, Ali I, *et al*. Improving influenza vaccine uptake in clinical risk groups: patient, provider and commissioner perspectives on the acceptability and feasibility of expanding delivery pathways in England. *BMJ Pub Health* 2024;2:e000929.

This article has been corrected since it was first published. Rajeka Lazarus has now been made a corresponding author.

