# Supplementary material for: Improving influenza vaccine uptake in clinical risk groups: patient, provider and commissioner perspectives on the acceptability and feasibility of expanding delivery pathways in England
Source: BMJ Public Health. 2024 Jun 28;2(1):e000929. doi: 10.1136/bmjph-2024-000929 (PMC11812908; doi:10.1136/bmjph-2024-000929)
Supplement: online supplemental file 1 [file bmjph-2-1-s001.pdf]

Participant Information Letter

Assessing the feasibility and acceptability of alternative seasonal flu vaccination delivery models for under-vaccinated clinical risk groups

Study information letter

October 2022

What is the purpose of the study?

The purpose of the study is to understand how uptake of the seasonal flu vaccination programme can be improved among under-vaccinated clinical risk groups. We are interested in learning about what influences decisions around the seasonal flu vaccine, and whether uptake of the vaccine could be improved by integrating delivery within existing use of services for chronic disease management. We are also keen to understand the feasibility of such delivery strategies.

What is involved in participating in the study?

A researcher will interview you about your experience of the seasonal flu vaccination programme in person or by using Zoom or Microsoft Teams. With your permission the interview will be recorded and transcribed.

Who is conducting the study?

The study is being conducted as part of a programme of research run by the London School of Hygiene & Tropical Medicine (LSHTM). Researchers from LSHTM work independently from UKHSA.

Who is funding the study?

This study is funded by the National Institute for Health Research.

Do I have to take part?

It is up to you to decide to take part in the study and the information provided in this sheet should help you decide. If you are interested in taking part, please email us your contact details and suggest times when it is best to reach you. A member of our team will then be in contact with you to explain the study in more detail giving you the opportunity to ask questions. If you agree to take part in the study, we will obtain your consent to allow us to take part in an interview. You are free to withdraw at any time, even during the interview, without giving a reason.

What will happen to me if I take part?

If you agree to take part you will be interviewed individually at a location that is conducive to privacy. These interviews will last between 45-60 minutes. With your permission, we will audio record the interview and it will then be transcribed by an approved transcription company.

Will my participation in the study be kept confidential?

Yes. Any information that you share with us during the course of the research will be kept strictly confidential and we will not tell anyone about your participation in this study. The interview

transcript will be made anonymous by removing names and references to places by using numerical identifiers and pseudonyms. Where appropriate, anonymised quotes from your interview may be used in publications or reports to illustrate certain points. Utmost care will be taken to ensure that you, your organisation and the area you work in cannot be identified through quotes or contextual information included in reports and publications. All personal data, apart from the consent form, will be deleted at the end of the study. The anonymised study data set will be stored for a minimum of 10 years. Journals, which publish findings from this study, may also ask us to deposit the anonymised data set in a data repository which is in the public domain.

What are the possible benefits of taking part?
Sharing your experiences around the seasonal flu vaccination programme will inform the delivery of services for eligible groups in the future.

What are the possible disadvantages and risks of taking part?
You may feel uncomfortable about talking about your experience but please be reassured that the aim of this study is to learn from your experience and not to evaluate you individually. The interviews will be conducted by researchers from the London School of Hygiene & Tropical Medicine. They do not have any professional ties with those responsible for providing the seasonal flu vaccination programme where you live, and they will respect your confidentiality and professional position.

Thank you very much for taking the time to read this information sheet.
Please contact us if you would like to find out more about the study.

Contacts:

Dr Ben Kasstan

Department of Global Health and Development
London School of Hygiene and Tropical Medicine
15-17 Tavistock Place
London, WC1H 9SH
Ben.kasstan@lshtm.ac.uk
