# Supplementary material for: Improving influenza vaccine uptake in clinical risk groups: patient, provider and commissioner perspectives on the acceptability and feasibility of expanding delivery pathways in England
Source: BMJ Public Health. 2024 Jun 28;2(1):e000929. doi: 10.1136/bmjph-2024-000929 (PMC11812908; doi:10.1136/bmjph-2024-000929)
Supplement: online supplemental file 2 [file bmjph-2-1-s002.pdf]

LONDON SCHOOL of HYGIENE &TROPICAL MEDICINE

Improving influenza vaccine uptake in clinical risk groups: Patient, provider and commissioner perspectives on the acceptability and feasibility of expanding delivery pathways in England

Topic Guide – clinical risk groups

Introduction and clarification of consent

Introduction: Introduce self

Aim: We are exploring the reasons for suboptimal uptake of seasonal influenza vaccination among eligible groups. Understanding how delivery of the seasonal influenza programme can be optimised will help to address inequalities in uptake.

Consent: Remind the participant of voluntary participation, audio recording, and anonymous quotes.

Switch audio recorder on: For the audio recording, can I confirm that you have read and understood the participant information sheet? Do you have any questions? Are you happy to take part in this interview?

Date of interview: ________________________

Interviewer name:__________________________

Participant anonymous number: _________________

Age: ____

Location of residence: _________________

Ethnicity: _________________

1. **Background information**

- Please tell me about yourself and the health conditions you receive care for.
    - Which secondary or non-primary care (not GP or pharmacy) services do you use to manage your health condition?

- Do conversations around general health, including risk of developing severe seasonal influenza, arise in your patient consultations in secondary and non-primary care

services?

2. Experiences of seasonal influenza

- Have you had seasonal influenza in the past? What symptoms did you have and how did you manage them?
    - Have you ever been admitted to hospital because of seasonal influenza?

- Do you consider influenza a serious risk? Does your chronic condition make illness from influenza more severe?

3. Understandings of seasonal influenza and vaccination

- Are you eligible to receive a seasonal influenza vaccination?

- Did your GP or practice nurse recommend that you have you have a seasonal influenza vaccine this year?

- Why did you not to take-up the seasonal influenza vaccine in the past 3 years?

- Have you had a seasonal influenza vaccine in the past?

- How accessible is the seasonal influenza vaccine for you?

- Did you feel informed about the possible benefits of the seasonal influenza vaccine, considering your health condition?
    - Were you concerned about side-effects?

4.Including influenza vaccination in patient care pathways

- How would you feel about having the seasonal influenza vaccine in the secondary or non-primary care services (e.g. NOT GP or pharmacy) you use to manage diabetes/liver disease/COPD/asthma?
    - How would you feel about having vaccination in in-patient versus out-patient services?
    - What benefits would this offer you?
    - Do you prefer to receive the seasonal influenza vaccine as part of primary care services?
    - Would receiving the influenza vaccine in secondary or community care services make the vaccine more accessible for you?

- Where would make most sense for you to receive the seasonal influenza vaccine?
    - e.g. Where in the patient pathway?

- What concerns would you have about seasonal influenza vaccination being delivered as part of your diabetes/liver disease/COPD/asthma care?

5.COVID-19 vaccine

- Did you receive any COVID-19 vaccines? Where did you receive them?
    - Did you receive the COVID-19 vaccine as part of the services you receive for diabetes/liver disease/COPD/asthma?
    - Can you think of any lessons from the COVID-19 vaccine programme to help improve the seasonal influenza vaccine programme?

6. Any other issues

Any other issues? Is there anything important that I have not asked you about?

(Reminder of prior questions. You mentioned X, Y Z. Would you be able to share that information with us?).

Thank them for their time and check preferences regarding receipt of summary of study findings.

Improving influenza vaccine uptake in clinical risk groups: Patient, provider and commissioner perspectives on the acceptability and feasibility of expanding delivery pathways in England

Topic Guide – healthcare providers (non-primary care settings)

Introduction and clarification of consent

Introduction: Introduce self

Aim: We are exploring the reasons for suboptimal uptake of seasonal influenza vaccination among eligible groups. Understanding how delivery of the seasonal influenza programme can be optimised will help to address inequalities in uptake.

Consent: Remind the participant of voluntary participation, audio recording, and anonymous quotes.

Switch audio recorder on: For the audio recording, can I confirm that you have read and understood the participant information sheet? Do you have any questions? Are you happy to take part in this interview?

Date of interview: ________________________

Interviewer name: _________________________

Participant anonymous code: _________________

Job title/role: ____

Location: _________________

1. **Background information**

- Please tell me about your current role and responsibilities as an HCP

2. Understandings of seasonal flu and vaccination

- Do conversations around general health, including seasonal influenza, arise in your consultations with patients (if applicable)?
- Are your patients eligible for the seasonal influenza vaccine?
- Does the burden of seasonal influenza affect patient use of services for diabetes/liver disease/COPD/asthma?

    - E.g. Do you recall your patients being unable to attend services/consultations because of seasonal influenza?

3.Including influenza vaccination in patient care pathways

- How feasible would it be to integrate seasonal influenza vaccines into secondary or non-primary care (e.g. not GP or pharmacy) you provide to your patients?
    - prompt – staff, funding, existing patient care provision?

- How feasible would it be to integrate seasonal influenza vaccines into in-patient versus out-patient services?

- How acceptable would it be to integrate seasonal influenza vaccine into secondary and non-primary care provision?

- How would you envisage seasonal influenza vaccination being delivered in your service?
    - e.g. where in the patient pathway?

- What concerns would you have about seasonal influenza vaccination being delivered to clinical risk group patients in your service?

- What obstacles could you envisage to seasonal influenza vaccination being delivered?

4. COVID-19 vaccination delivery

Was the COVID-19 vaccination programme delivered in your clinic or integrated into patient pathways?

- If so, can you tell us how the delivery of COVID-19 vaccines worked in practice?
- Could the delivery of COVID-19 vaccines inform delivery of the seasonal influenza vaccination programme? How?

5. Any other issues

Any other issues? Is there anything important that I have not asked you about?

(Reminder of prior questions. You mentioned X, Y Z. Would you be able to share that information with us?).

Topic Guide – healthcare providers (primary care)

Introduction and clarification of consent

Introduction: Introduce self

Aim: We are exploring the reasons for suboptimal uptake of seasonal influenza vaccination among eligible groups. Understanding how delivery of the seasonal influenza programme can be optimised will help to address inequalities in uptake.

Consent: Remind the participant of voluntary participation, audio recording, and anonymous quotes.

Switch audio recorder on: For the audio recording, can I confirm that you have read and understood the participant information sheet? Do you have any questions? Are you happy to take part in this interview?

Seasonal influenza vaccine: service providers and commissioners

Date of interview: ________________________

Interviewer name: _________________________

Participant anonymous code: _________________

Job title/role: ____

Location: _________________

1. **Background information**

- Please tell me about your current role and responsibilities as an HCP

2. Understandings of seasonal flu and vaccination

- Can you tell us about how seasonal influenza vaccine is delivered in your practice ?
  - Who does what?
  - Call and recall system
  - Opportunistic vaccination

- Outside of the influenza season, do conversations around general health, including seasonal influenza, arise in your consultations with patients?

- We will now talk about patients in clinical groups below 65?

    - Do you know what share of these patients do not get vaccinated in your practice?

- In your experience, what are the reasons why these patients do not receive the influenza vaccine?

COVID-19 vaccination delivery

- Was the COVID-19 vaccination programme delivered in your clinic or integrated into patient pathways?

- If so, can you tell us how the delivery of COVID-19 vaccines worked in practice?

- Could the delivery of COVID-19 vaccines inform delivery of the seasonal influenza vaccination programme? How?

3.Improving influenza vaccination in patient care pathways

- What would be the most effective strategy to increase uptake of these patients at primary care level in your view?

- What would help you?

4. Working with secondary care to increase uptake

- Do you see an opportunity to work in collaboration with secondary care providers to improve influenza vaccine uptake in clinical risk groups <65 and how?

- How feasible would it be to integrate seasonal influenza vaccines into secondary or non-primary care (e.g. not GP or pharmacy) you provide to your patients in the case they do not attend your practice?

- What concerns would you have about seasonal influenza vaccination being delivered to clinical risk group patients in secondary care settings?

- What obstacles could you envisage to seasonal influenza vaccination being delivered in secondary care settings?

5. Any other issues

Any other issues? Is there anything important that I have not asked you about?

(Reminder of prior questions. You mentioned X, Y Z. Would you be able to share that information with us?).

Thank them for their time and check preferences regarding receipt of summary of study findings.

Topic Guide – Commissioners

Introduction and clarification of consent

Introduction: Introduce self

Aim: We are exploring the reasons for suboptimal uptake of seasonal influenza vaccination among eligible groups. Understanding how delivery of the seasonal influenza programme can be optimised will help to address inequalities in uptake.

Consent: Remind the participant of voluntary participation, audio recording, and anonymous quotes.

Switch audio recorder on: For the audio recording, can I confirm that you have read and understood the participant information sheet? Do you have any questions? Are you happy to take part in this interview?

Seasonal influenza vaccine: service providers and commissioners

Date of interview: ________________________

Interviewer name: _________________________

Participant anonymouse code: _________________

Job title/role: ____

Location: _________________

1. **Background information**

- Please tell me about your current role and responsibilities

2. Understandings of seasonal influenza and vaccination

- Can you tell us about your commissioning strategy for influenza vaccination in clinical risk groups <65 year old?
    - Overall strategy
    - Communication strategy
    - Call and recall systems
    - Opportunistic vaccination

- What do you think are the reasons why people under the age of 65 in at risk groups do not receive the influenza vaccine?

COVID-19 vaccination delivery

- Has the COVID-19 vaccination programme delivered changed in any way the way the seasonal influenza programme is delivered- including joint delivery.

- How has the delivery of COVID-19 vaccines informed delivery of the seasonal influenza vaccination programme (eg undervaccinated groups) in terms of communication/ delivery model, community engagement?

3.Improving influenza vaccination in patient care pathways

- What would be the most effective strategy to increase uptake of these patients at primary care level in your view?

- What would help?

4. Working with secondary care to increase uptake

- Do you see an opportunity in working in collaboration with secondary care providers to improve influenza vaccine uptake in clinical risk groups and how?

- How feasible would it be to integrate seasonal influenza vaccines into secondary or non-primary care (e.g. not GP or pharmacy) you provide to your patients in the case they do not attend your practice?

- If you have commissioned such services, could you explain how effective it has been and challenges that arose?

- What concerns would you have about seasonal influenza vaccination being delivered to clinical risk group patients in secondary care settings?

- What obstacles could you envisage to seasonal influenza vaccination being delivered in secondary care settings?

5. Any other issues

Any other issues? Is there anything important that I have not asked you about?

(Reminder of prior questions. You mentioned X, Y Z. Would you be able to share that information with us?).

Thank them for their time and check preferences regarding receipt of summary of study findings.
